# Validation of a multi-residue method to determine deltamethrin and alpha-cypermethrin in mosquito nets by gas chromatography with electron capture detection (GC-μECD)

**DOI:** 10.1186/1756-3305-6-77

**Published:** 2013-03-20

**Authors:** Jean Pierre Nabléni Ouattara, Olivier Pigeon, Pieter Spanoghe

**Affiliations:** 1 Department of Crop Protection, Laboratory of Crop Protection Chemistry, Ghent University, Coupure links 653, Gent B-9000, Belgium; 2 Walloon Agricultural Research Centre (CRA-W), Agriculture and Natural Environment Department, Plant Protection and Biocides Physico-Chemistry and Residues Unit, Rue du Bordia, 11, Gembloux, B-5030, Belgium; 3 Laboratoire National de Santé Publique (LNSP), Boulevard des Tansoba kiéma, Ouagadougou 09 09 BP 24, Burkina Faso

**Keywords:** Analytical method validation, Mosquito net, Deltamethrin, Alpha-cypermethrin, GC-μECD

## Abstract

**Background:**

Nowadays long-lasting insecticidal mosquito nets (LNs) are frequently used around the world to protect people against malaria vectors. As they contain insecticide, laboratory control is needed to check whether the content of the active ingredient follows the conditions of the manufacturer and also if the active ingredient is still present after some time of use. For this purpose, an analytical method had to be developed. The fact that LNs include a range of polymers for the yarn and use coated or incorporated technologies for the active ingredient, it is a challenge to find only one analytical method determining the active ingredient in LNs, which takes into account both impregnation technologies. Some methods are provided by international organizations but are limited by the determination of only one pesticide per method. The aim of this study was to optimize a short time extraction method for deltamethrin and alpha-cypermethrin from coated and incorporated mosquito nets and also to detect both insecticides in one analytical run, using gas chromatography with electron capture detection (GC-μECD).

**Methods:**

Based on the literature, the most suitable solvent and the adequate extraction process for the insecticides used for net making were identified and adapted for the new multi-residue method.

**Results:**

The validation data of the multi-residue method to determine deltamethrin and alpha-cypermethrin in mosquito nets by GC-μECD are given. Depending on the concentration of the active ingredient spiked on the nets, the mean recovery for alpha-cypermethrin ranged between 86% and 107% with a relative standard deviation below 3.5%. For deltamethrin it ranged between 90% and 108% with a relative standard deviation also below 3.5%. The limit of detection is 0.009 g.a.i/kg of net (0.3 mg a.i./m^2^ of net) both for alpha-cypermethrin and deltamethrin.

**Conclusions:**

Data obtained are excellent. A 30 minutes reflux extraction method with xylene was developed to determine alpha-cypermethrin and deltamethrin in long-lasting insecticidal mosquito nets (LNs) by gas chromatography with electron capture detection (GC-μECD). The method can be easily extended to others pyrethroid used for mosquito net treatment. This paper also presents an overview of the studies dealing with pesticide determination in mosquito nets.

## Background

Long-lasting insecticidal mosquito nets (LNs) become more and more important in the control and prevention of diseases like malaria. They are considered by the Food and Agriculture Organization (FAO) of the United Nations and World Health Organization (WHO) Manual on pesticides specifications as formulations, because they contain insecticides [1]. So they must fit some requirements for regulatory monitoring, import/export certification and risk assessment. For this purpose, a good analytical method must be available for the determination of the active ingredient. The International Organization for Standardization and the International Electrotechnical Commission (ISO/IEC) through the ISO/IEC 17025:2005 [2] asks to validate the test and analytical methods when they are not standardized or when standardized methods are used outside their scope. In this way, the Collaborative International Pesticides Analytical Council (CIPAC) [3] that promotes the international agreement on methods for the analysis of pesticides and physico-chemical test methods of formulations provides some selected methods in order to meet the urgent need to characterize LN. The fact that LNs include a range of polymers for the yarn and use coated or incorporated technologies for the active ingredient, it seems, even if harmonized analytical methods as provided by CIPAC are used, that technological variations within LN types are too large to cover the determination of the active ingredient content with one method. So a good compromise has to be found between the need to harmonize analytical methods for quality control purpose and the need to establish accurate specifications suitable for each type of LN product [4]. Four methods have been developed by CIPAC for the determination of active ingredient content in LNs [5-8]. The CIPAC methods and some methods published in the literature determine the insecticide content of LN by High-Performance Liquid Chromatography with UV Diode-Array Detection (HPLC-DAD), High Performance Thin Layer Chromatography (HPTLC), Gas Chromatography with Flame Ionization Detection (GC-FID) or Gas Chromatography with Electron Capture Detection (GC-ECD) [5-10]. They are applicable for the determination of only one pesticide per method. If the main limit is the fact that this is only one active ingredient determination by method, the GC-FID and HPLC–DAD methods seem to be very suitable for assessment of the baseline dose of LNs and are used by most manufacturers for quality control purposes [10]. Their main drawback is the lack of detector sensitivity [11]. For this reason, when the interest is to determine the residues or remaining amount of insecticide on or into the net after some time, more sensitive methods like gas chromatography with electron capture detection (GC-ECD) are required [10].

To cover the analysis of all the types of nets (coated or incorporated, different insecticides, baseline dose or residue amount of insecticides), a laboratory today needs to use several analytical methods.

The drawbacks of using one single method to determine only one insecticide are the high demand of manpower, solvents, equipments and laboratory space. There is an urgent need to develop more cost-effective analytical procedures [11]. The challenge here was to find a multi-pesticide method for the determination of insecticides in different types of LNs.

The goal of this study was to optimize a short time extraction method for deltamethrin and alpha-cypermethrin in coated and incorporated mosquito nets and also to detect both insecticides in one analytical run using GC-μECD with a high sensitivity.

## Methods

### Literature search

A literature search on pesticides determination in mosquito nets was carried out on the ISI Web of Knowledge data and on the CIPAC web site. The following combinations of keywords were used for the search; Mosquito nets, analytical method, validation, pesticide, deltamethrin, alpha-cypermethrin, chromatography analysis. Only the articles in open literature relevant to our work were kept. Reference sections within the articles obtained were used to find more studies that might have been missed during the general search on Web of Knowledge. Based on the literature, a new multi-pesticide method has been proposed. Details of the method are presented here after.

### Chemicals and reagents

Xylene analytical grade reagent was purchased from Sigma-Aldrich Logistik GmbH, Germany.

Deltamethrin standard with 99% purity and alpha-cypermethrin standard with 97.5% purity were purchased from Dr. Ehrenstorfer GmbH.

Deltamethrin technical (99.2%) was purchased from Bayer CropScience. Alpha-cypermethrin technical (100.0%) was given by BASF Chemical Company.

## Materials

A net made with polyester without any insecticide was given by Utexbel S.A. and was called blank sample.

The two impregnation technologies of mosquito nets were analyzed for the evaluation of the proposed method. Table 1 gives an overview of the specifications of the manufacturers and the characteristics of the used insecticides and impregnation of the nets.

**Table 1:** Characteristics of LNs provided by the manufacturer

**Net type**	**Technology**	**Company**	**Material**		**Active ingredient**	
			**Fabric**	**Weight (g/m**^**2**^**)**	**Insecticide**	**Content** (**mg/m**^**2**^**)**
Interceptor^®^	Coated	BASF Chemical Company	100% polyester multifilament	40	alpha-cypermethrin	200
PermaNet^®^2.0	Coated	Vestergaard Frandsen SA	100% polyester multifilament	30	deltamethrin	55
Netprotect^®^	Incorporated	Dean Superior Textile Co.	100% Polyethylene	44	deltamethrin	79

### Preparation of calibration solutions

#### Stock solution

Individual stock solutions of 500 μg/mL of deltamethrin and alpha-cypermethrin were prepared in xylene. From the individual stock solutions, a mixture stock solution of deltamethrin and alpha-cypermethrin was prepared in xylene at 50 μg/mL.

#### Calibration solutions

From of the mixture stock solution, different concentration levels (0.01, 0.1, 0.5, 1, 3, 5, 7 μg/mL) were prepared by appropriate dilution with xylene to form the calibration curve solutions.

### Sample preparation

#### Fortification solutions

Three concentration levels of fortification solutions were prepared with pesticides technical materials. The high level of fortification solution with concentration of 1200 μg/mL of deltamethrin and 3000 μg/mL of alpha-cypermethrin was prepared by weighting on an analytical balance the amount of the respective technical product and by diluting it in 100 mL of xylene. Two other concentrations were prepared by appropriate dilution of the first solution with xylene. The concentration of the middle level of fortification solution was 600 μg/mL of deltamethin and 1500 μg/mL of alpha-cypermethrin. The concentration of the low level fortification solution was 60 μg/mL of deltamethin and 150 μg/mL of alpha-cypermethrin.

#### Sample spiking

The blank net was cut in small pieces with a clean scissor. 20 portions of 300 ± 0.1 mg of each were weighed into different flat-bottom boiling flasks of 100 mL. The samples were divided in three groups named the low spiked samples (L) (7 samples), the middle spiked samples (M) (7 samples) and the high spiked samples (H) (6 samples). Each sample of group L was spiked with 1 mL of the low level fortification solution. Samples of group M were spiked with 1 mL of the middle level fortification solution and samples of group H were spiked with 1 mL of the high level fortification solution. The fortified or spiked samples stood alone for 30 minutes to allow the active ingredient to interact with the matrix before the extraction process starts.

The required fortification levels related to the 3 groups were: for group L 0.2 g a.i./kg net (deltamethrin) and 0.5 g a.i./kg net (alpha-cypermethrin), for group M 2 g a.i./kg net (deltamethrin) and 5 g a.i./kg net (alpha-cypermethrin) and for group H 4 g a.i./kg net (deltamethrin) and 10 g a.i./kg net (alpha-cypermethrin).

### Reflux extraction

40 mL of xylene was added to the fortified sample. The flat-bottom boiling flask was connected to a reflux condenser then heated and stirred with Heidolph MR 3001 to reflux for 30 minutes. The extract solution was cooled to ambient temperature and filtered through a büchner filter funnel using whatman™ filter paper into a 50 mL volumetric flask. The filtration cake was rinsed and the extract solution was extended to 50 mL with xylene. After that, 1 mL of the extract solution was diluted into 10 mL of xylene and a portion of this solution was transferred into an injection vial.

### Apparatus and GC analysis

**Figure 1:**
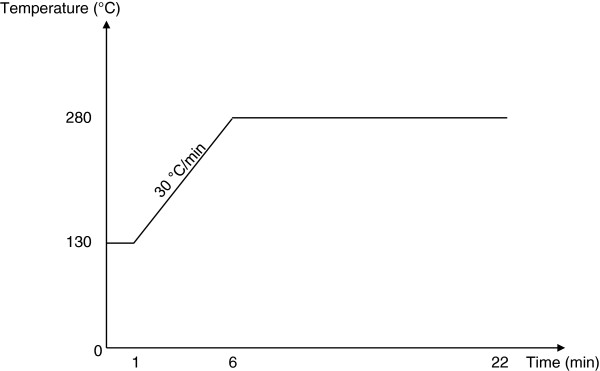
Oven temperature program during analysis.

Samples were analyzed with GC-μECD Agilent Technologies 6890 N equipped with an auto sampler Agilent Technologies 7683 Series injector which was used in split mode. The chromatographic separation was performed on a HP-5 (5% Phenyl Methyl Siloxane) capillary column (30 m x 0.250 mm i.d., 0.25 μm film thickness). Helium was used as the carrier gas and kept at constant pressure of 102.7 kPa with a nominal flow of 0.9 mL/min. The split ratio, split flow and total flow were respectively 50:1, 45.5 mL/min and 49.9 mL/min. The μECD detector temperature was 300°C with nitrogen as make-up gas kept at constant flow of 60.0 mL/min. For each sample two chromatographic injections were done and the mean was reported as mass of active ingredient per unit mass of netting (g/kg). The injection volume was 1 μL and the oven temperature was programmed as: isothermal at 130°C for 1 minute, from 130°C to 280°C at 30°C/minute and held for 16 minutes (Figure 1).

Others equipments used in this study were a calibrated Sartorius LA 230P analytical balance to weight standards, technical products and samples, a Heidolph MR 3001 to heat and stir during the extraction process.

### Analytical performance

#### Selectivity/specificity

The selectivity of a method refers to the extent to which it identifies particular analyte(s) in a complex mixture without interference from other components in the mixture [12]. To test selectivity, the individual standard solutions were injected, followed by the mixture standard solutions to check whether interference exists.

A blank sample was extracted in 3 replicates following the analytical method to check the absence of interference peaks under the same conditions with regard to degradation products, impurities and the matrix. A reagent blank was also made and all the steps of the extraction process were followed to check whether there were interference peaks from solvents and consumables used and the active ingredients.

#### Linearity of the detector response

The linearity was performed for deltamethrin and alpha-cypermethrin. Seven concentration levels (0.01, 0.1, 0.5, 1, 3, 5, 7 μg/mL) determining the calibration curve solutions were injected twice. The linearity of each active ingredient was tested using the ordinary linear regressions of the calibration curve and the coefficient of determination (R^2^) was calculated.

#### Precision/ Repeatability

The precision of an analytical procedure expresses the closeness of agreement (degree of scatter) between a series of measurements obtained from multiple sampling and analysis of the same homogeneous sample under the prescribed conditions [12]. As a simple assessment of repeatability is acceptable to express the precision, the repeatability was evaluated by the coefficient of variation of the measurement from each spiking level and for each active ingredient. Repeatability is the closeness of agreement between mutually independent test results obtained with the same operator using the same equipment within short intervals of time [13].

#### Recovery/accuracy

The accuracy of a method is the closeness of the measured value to the true value for the sample [14]. The accuracy was evaluated by using the spiked-placebo recovery method. Blank samples were spiked at 3 levels and analyzed under the same conditions (same day same operator) and the ratio of the calculated amount to the expected amount expressed as a percentage was used to assess the recovery. The evaluation of the proposed method was also done by analyzing 3 commercial nets.

#### Limits of detection / quantification

The limit of detection (LOD) is the lowest amount of an analyte in a sample that can be detected. It was determined using the calibration data as following [15]:

Step 1: Calculate the upper confidence limit for the intercept (CLa) of the linear regression equation (Y = a +bX) for the calibration. CL_a_ = a + *t*_*α,n-2*_ x *S*_*a*_

Step 2: Compute the corresponding analyte concentration of limit of decision (CC_α_) as: CC_α_ = (CL_a_ - a)/b

Step 3: Compute the LOD as LOD = CC_α_ + *t*_*β,n-2*_ x *SC*_*B*_

Where *t*_*α,n-2*_*, and t*_*β,n-2*_ are the one tailed Student‘s *t* values for n-2 degrees of freedom; α and β are selected as equal to 0.1 level; *S*_*a*_ is the standard deviation of the intercept; *SC*_*B*_ the standard deviation of CC_α_.

The limit of quantification (LOQ) was obtained from limit of detection by LOQ = 10/3 x LOD [16].

## Results and discussion

### Literature review of available methods

Published and grey literature has been sought and reviewed to obtain available information on analysis of pesticides on mosquito nets. The Table 2 summarizes the database search and presents details of the analytical methods.

**Table 2:** Overview of studies dealing with pesticide residue determination in mosquito nets

	**Pesticides**	**Nets**^**a**^	**Extraction principle**	**Equipment**	**Analytical data**		
	**&**				**Range**	**Recovery**	**LOQ**
	**Publications**						
1	Alpha-cypermethrin [8]	LNs	**Extraction solvent:**	GC-FID	Not available		
			Tetrahydrofuran				
			**Process:**				
			Reflux at 90°C (oil bath temperature) during 5 min				
2	Alpha-cypermethrin [17]	Coated LNs	**Extraction solvent:**	GC-FID	0.05 – 10 g/kg	98 – 101% with RSD < 5%	0.05 g/kg
			Tetrahydrofuran				
			**Process:**				
			Reflux at 90°C during 5 min				
3	Alpha-cypermethrin [8,17]	Incorporated LNs	**Extraction solvent:**	GC-FID	0.05 – 10 g/kg	99 – 101% with RSD < 5%	0.05 g/kg
			Xylene				
			**Process:**				
			Reflux at 130°C during 30 min				
4	Alpha-cypermethrin [18]	ITNs	**Extraction solvent:**	GC-ECD	Not available		
			Xylene				
			**Process:**				
			Reflux during 60 min				
5	Alpha-cypermethrin [19]	ITNs	CIPAC protocol for extracting alpha-cypermethrin	HPLC	Not available		
6	Alpha-cypermethrin [9]	LNs	**Extraction solvent:**	HPTLC-UV	Not available		
			Acetone				
			**Process:**				
			Shake during 10 min				
7	Deltamethrin [6]	Coated LNs	**Extraction solvent:**	HPLC-DAD	-	99.7% (95% CI 98.6 - 101.6%)	-
			Iso-octane + 1.4 dioxane (80/20) + 0.15% HPLC water grade				
			**Mobile phase:**				
			Iso-octane + 1,4 dioxane (95/5) **Process:**				
			Ultrasonic bath at 80°C for 15 min + shake at room temperature at the speed of 155 beats per minute during 30 min				
8	Deltamethrin [7]	LNs	**Extraction solvent:**	HPLC-DAD	Not available		
			Xylene into a reflux flask				
			**Mobile phase:**				
			Iso-octane + 1,4 dioxane (+0.15% water) (94/6)				
			**Process:**				
			Reflux during 30 min under stirring Reconstitution of the extract solution in iso-octane + 1,4 dioxane (94/6)				
9	Deltamethrin [20-23]	ITNs	**Extraction solvent:**	HPLC-DAD	0 - 0.4 μg/mL (standard curve range)		
			Acetonitrile				
			**Mobile phase:**				
			Water/acetonitrile (90/10% v/v)				
10	Deltamethrin [24]	Permanet2.0	**Extraction solvent:**	HPLC-UV	No available		
			Acetone and acetonitrile				
			**Mobile phase:**				
			Methanol/water (90/10)				
			**Process:**				
			Vortexing				
11	Deltamethrin [25]	Coated LNs	**Extraction solvent:**	HPLC-DAD	0.01 – 4 g/kg	93 - 99% with RSD < 5%	0.01 g/kg
			Iso-octane + 1.4 dioxane (80/20)				
			**Mobile phase:**				
			Iso-octane + 1,4 dioxane (+ 0.15% water) (94/6, v/v)				
			**Process:**				
			Ultrasonic bath at 70°C for 15 min + shaking at ambient temperature at the speed of 150-200 beat per minute during 30 min				
12	Deltamethrin [25]	Coated LNs	**Extraction solvent:**	GC-FID	0.01 – 4 g/kg	93 - 104% with RSD < 5%	0.01 g/kg
			Iso-octane + 1.4 dioxane (80/20)				
			**Mobile phase:**				
			Iso-octane + 1,4 dioxane (+ 0.15% water) (94/6, v/v)				
			**Process:**				
			Ultrasonic bath at 70°C for 15 min + shaking at ambient temperature at the speed of 150-200 beat per minute during 30 min				
13	Deltamethrin [26-29]	LNs	**Extraction solvent:**	GC-FID	0.01 – 20 g/kg	93 - 110% with RSD < 5%	0.01 g/kg
			Xylene				
			**Process:**				
			Reflux during 60 min				
14	Deltamethrin [10,30,31]	ITNs	**Extraction solvent:**	GC-ECD	0.05 – 150 mg/m^2^	95 - 102% with RSD between 7 - 11%	0.05 mg/m^2^
			Xylene				
			**Process:**				
			Reflux during 60 min				
15	Deltamethrin [32,33]	ITNs	**Extraction solvent:**	GC-ECD	Not available		
			Acetone				
			**Process:**				
			Sonication for 30 min and standing overnight				
16	Permethrin [5]	LNs	**Extraction solvent:**	GC-FID	Not available		
			Heptane				
			**Process:**				
			Stand in a water bath at 85-90°C for 45 min and shake once or twice during this time				
17	Permethrin [32]	ITNs	**Extraction solvent:**	GC-FID	Not available		
			Chloroform				
			**Process:**				
			Sonication for 30 min and standing overnight				
18	Deltamethrin, Cyfluthrin, Permethrin, Etofenprox [34]	ITNs	**Extraction solvent:**	GC-MSD	Not available		
			Toluene				
			**Process:**				
			Stirring during 10 min followed by ultra sonication 10 min				
19	Permethrin [35]	ITNs	**Extraction solvent:**	GC-MSD	Not available		
			Ethanol				
			**Process:**				
			Stirring during 10 min followed by ultra sonification 10 min				
20	Lambda-cyhalothrin [36]	Coated LNs	**Extraction solvent:**	GC-FID	Not available		
			Acetone + glacial acetic acid (95/5)				
			**Process:**				
			Sonification for 30 min followed by swirling 15 min				
21	Piperonyl butoxide [37]	LNs	**Extraction solvent:**	GC-FID	Not available		
			Xylene				
			**Process:**				
			Reflux during 30 min				

^**a**^ Abbreviations for nets types**:***ITN* = Insecticide-treated net; a mosquito net that has been treated by dipping in a WHO-recommended insecticide [38]. *LN* = Long Lasting Insecticidal Net; a factory-treated mosquito net made with netting material that has insecticide incorporated within or bound around the fibers [38].

Most studies report analytical methods for the detection of pyrethroids. This confirms the fact that pyrethroids are the class of insecticides mostly used for the impregnation of mosquito nets [39]. Most methods handle the detection of alpha-cypermethrin, deltamethrin, cyfluthrin or permethrin. The major solvents to extract the pesticides out of the nets are xylene or a mixture of Iso-octane + 1,4 dioxane. A GC-ECD, GC-FID or HPLC-DAD seems the most frequently used analytical equipment and detection method. Concerning what is given in literature on validation data of the different proposed methods, the lack of information is really remarkable. It was also seen that among the analytical methods found, most of them determine only one pesticide using one analytical method.

A method using reflux extraction with xylene seems to be the most suitable for pyrethroids extraction from LNs. According to Kilian et al., [10] who checked the correlation between analytical protocols for the determination of deltamethrin, the GC-ECD analytical technique is the most universally applicable method for the determination of insecticides in LN. A GC-ECD is even able to detect very small amounts of insecticide [10].

### Validation of the selected method

#### Selectivity / Specificity

**Figure 2:**
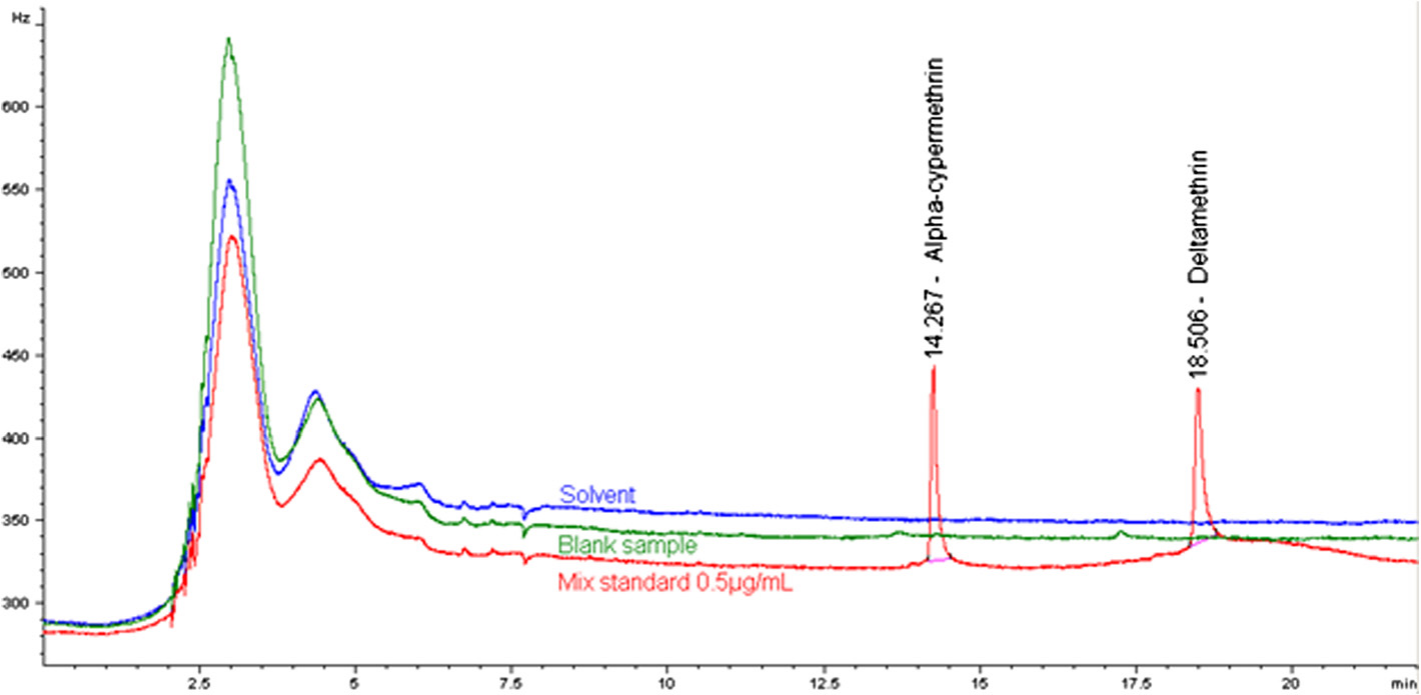
Combined chromatograms for evaluation of the specificity.

A blank net was analyzed following the analytical protocol to check the interference with alpha-cypermethrin and deltamethrin peaks. No peak appeared in the blank at the retention time of alpha-cypermethrin and deltamethrin. The reagent blank (xylene) did not show any interference with the mix standard solution (Figure 2).

**Figure 3:**
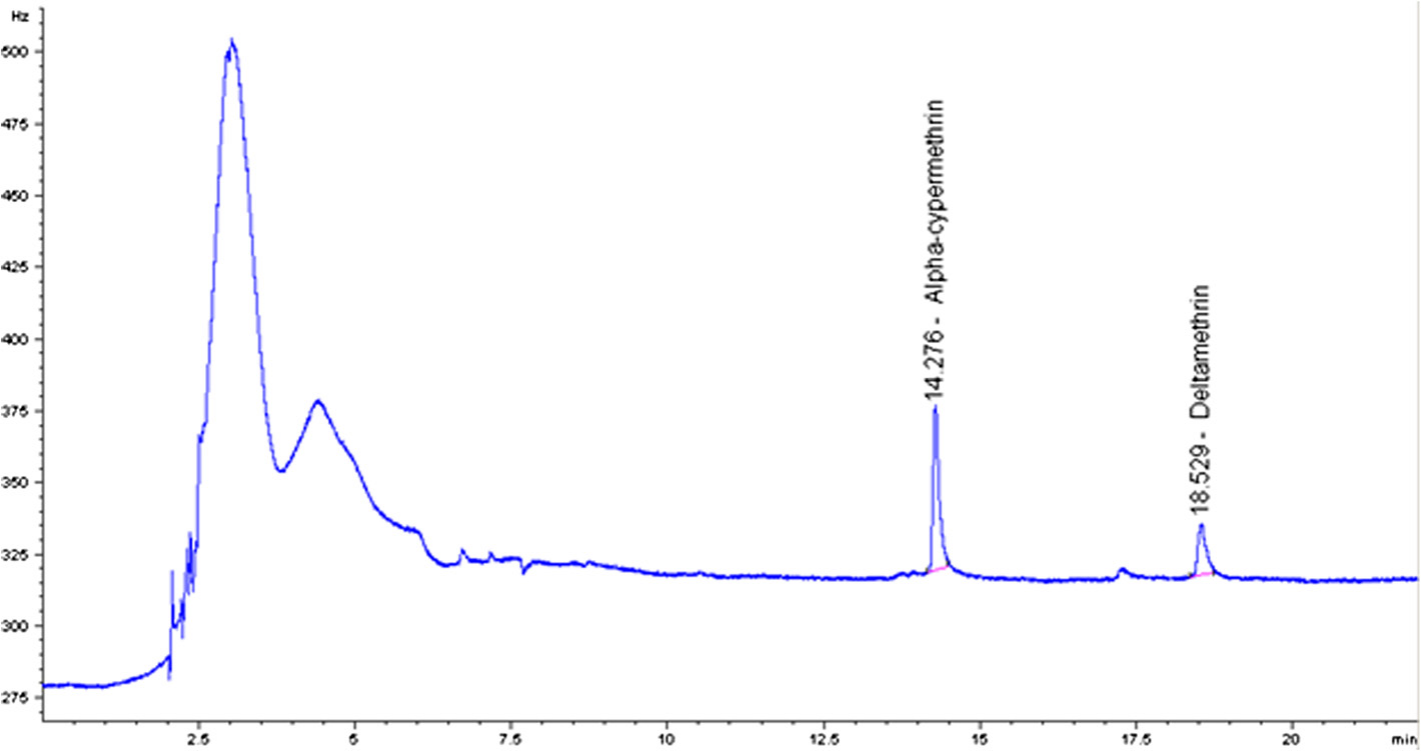
Chromatogram of the low dose spiked sample.

The injection of the low spiked (Figure 3) sample extract solutions showed a good separation of the deltamethrin and alpha-cypermethrin peaks without any interference.

This observation points out that the extraction method seems to be selective and free from positive interference of co-extracted compounds, at least at the retention time window of interest.

#### Linearity of the detector response

Standard solutions determining the calibration curves were injected and following the ordinary linear regression, a regression equation of *Y* = 1861 *X – 143 for alpha-cypermethrin and of *Y* = 2119 * *X* – 166 for deltamethrin was obtained; with *X* as concentration expressed in μg/mL and *Y* as the peak area. The coefficients of determination (R^2^) values were greater than 0.9955 (0.9992 and 0.9993 respectively for alpha-cypermethrin and deltamethrin) with +1 as Pearson’s correlation coefficient (r) for both active ingredients. This showed that there was a very strong correlation between the increase in peak area and the increase in concentration of the compounds, the higher the R^2^ or r, the stronger the relationship is.

#### Repeatability / recovery

The results in Table 3 showed that depending on concentration of active ingredient spiked on the nets, the mean recovery ranged between 86% and 107% for alpha-cypermethrin with relative standard deviation below 3.5%. For deltamethrin it ranged between 90% and 108% with relative standard deviation also below 3.5%. The Table 3 showed also that, according to the acceptable criteria, the mean recovery and the RSD obtained are good. The observed RSD values were lower than those found in literature (7 and 11%) [10].

**Table 3:** Analytical data for the low, middle and high dose spiked samples

**Spiking level**		**Alpha-cypermethrin 0.5 g a.i./kg of net**		**Deltamethrin 0.2 g a.i./kg of net**	
		**Amount (g/kg)**	**Recovery (%)**	**Amount (g/kg)**	**Recovery (%)**
Low spiked sample^b^ n=7	L1	0.424	85.18	0.180	90.33
	L2	0.438	88.39	0.181	91.44
	L3	0.415	84.48	0.176	89.64
	L4	0.428	85.85	0.175	87.95
	L5	0.412	83.75	0.178	90.58
	L6	0.445	89.09	0.181	90.65
	L7	0.419	85.81	0.177	90.72
	**L Mean**	0.426	**86.08**	0.178	**90.19**
	SD	0.01	1.97	0.002	1.12
	**RSD %**	2.83	**2.29**	1.317	**1.24**
	**Acceptable mean recovery**[12]		**80 - 120**		**80 - 120**
	**Acceptable RSD**[12]		**≤ 10**		**≤ 10**
		**Alpha-cypermethrin 5 g a.i./kg of net**		**Deltamethrin 2 g a.i./kg of net**	
Middle spiked sample n=7	M1	5.230	106.06	2.007	101.90
	M2	5.466	110.38	2.088	105.56
	M3	5.158	104.59	1.953	99.13
	M4	5.290	108.26	2.026	103.79
	M5	5.170	104.20	1.991	100.42
	M6	5.543	112.20	2.117	107.27
	M7	5.109	104.18	1.948	99.41
	**M Mean**	5.281	**107.12**	2.019	**102.50**
	SD	0.16	3.22	0.06	3.14
	**RSD %**	3.12	**3.01**	3.19	**3.07**
	**Acceptable mean recovery**[12]		**90 - 110**		**90 - 110**
	**Acceptable RSD**[12]		**≤ 5**		**≤ 5**
		**Alpha-cypermethrin 10 g a.i./kg of net**		**Deltamethrin 4 g a.i./kg of net**	
High spiked sample n=6	H1	9.977	101.39	4.141	105.33
	H2	10.034	100.14	4.225	105.54
	H3	10.073	101.17	4.199	105.56
	H4	10.099	102.27	4.470	113.31
	H5	9.802	99.39	4.297	109.06
	H6	9.724	97.79	4.286	107.89
	**H Mean**	9.951	**100.36**	4.270	**107.78**
	SD	0.15	1.61	0.11	3.10
	**RSD %**	1.55	**1.61**	2.67	**2.88**
	**Acceptable mean recovery**[12]		**98 - 102**		**90 - 110**
	**Acceptable RSD**[12]		**≤ 2**		**≤ 5**

^b^ Active ingredient concentrations in low spiked samples were calculated with reduced calibration curve (0.01, 0.1, 0.5 μg/mL).

#### Evaluation of the method on commercial nets

Samples provided by 3 manufacturers of LNs were taken randomly for analysis. The results in Table 4 showed that the recoveries were more than 75 % (94, 99 and 80% respectively for Interceptor^®^, PermaNet^®^2.0 and Netprotect^®^). The concentration of the insecticides into the LNs were slightly different compared to the target of 5 g/kg (200 mg/m^2^) for Interceptor^®^ nets, 1.83 g/kg (55 mg/m^2^) for PermaNet^®^2.0 nets and 1.8 g/kg (79 mg/m^2^) for Netprotect^®^ but within the specifications of the manufacturers which is ± 25% [1].

**Table 4:** Active ingredient recovery from coated and incorporated nets samples

**Samples**	**Interceptor^®^**		**PermaNet^®^2.0**		**Netprotect^®^**	
	**Coated**		**Coated**		**Incorporated**	
	**Alpha-cypermethrin**		**Deltamethrin**		**Deltamethrin**	
	**g a.i./kg of net**	**mg a.i./m**^**2 **^**of net**	**g a.i./kg of net**	**mg a.i./m**^**2 **^**of net**	**g a.i./kg of net**	**mg a.i./m**^**2 **^**of net**
S1	4.20	168	1.84	55	1.48	65
S2	4.31	172	1.94	58	1.53	67
S3	5.66	226	2.20	66	1.44	63
S4	5.10	204	1.58	47	1.42	62
S5	4.26	170	1.53	46	1.37	60
**Mean**	**4.71**	**188**	**1.82**	**55**	**1.45**	**64**
**Recovery (%)**	**94**		**99**		**80**	

#### Limits of detection (LOD) and of quantification (LOQ)

The LOD of the method for each active ingredient was determined from calibration curves data [15]. The calculations were done using a statistics template made by Ambrus Arpad from International Atomic Energy Agency (IAEA) Agricultural Unit of Seiberdorf [15]. Then LOQ was evaluated as 10/3 times the LOD. The Table 5 shows the LOD and LOQ values.

**Table 5:** Values of LOD and LOQ

	**alpha-cypermethrin**		**deltamethrin**	
	**g a.i./kg of net**	**mg a.i./m**^**2 **^**of net**	**g a.i./kg of net**	**mg a.i./m**^**2 **^**of net**
LOD	0.0094	0.282	0.0088	0.264
LOQ	0.031	0.930	0.029	0.870

## Conclusions

A 30 minutes reflux extraction method with xylene was developed to determine alpha-cypermethrin and deltamethrin in long-lasting insecticidal mosquito nets (LNs) by gas chromatography with electron capture detection (GC-μECD). This study confirmed the fact that a GC-ECD protocol is suitable for insecticide determination on coated as well as on incorporated LNs [10]. The selectivity and specificity of the method has been demonstrated as the data showed the absence of interference peaks with regard to degradation products, impurities and the matrix. The chromatographic conditions showed also a good separation between deltamethrin and alpha-cypermethrin peaks.

The linearity of the detector response was fine for all the compounds as the coefficient of determination (R^2^) was more than 0.9955. The LODs, the range of recoveries and RSD values were satisfying. The method is able to determine low amounts of the insecticide without interference peaks. So this method can be used for quality control and also for research programs where the interest is to determine the remaining amount of insecticide from used LNs.

## Competing interests

The authors declare that they have no competing interests.

## Authors’ contributions

JPNO designed the study, carried out the study, analyzed the data and drafted the manuscript. OP helped to design the study, assured data quality and critically revised the manuscript. PS helped to conceive of the study, analyzed the data and critically revised the manuscript. All authors read and approved the final manuscript.
